# Comparison of Outcome Measures for Traditional and Online Support Groups for Breast Cancer Patients: An Integrative Literature Review

**Published:** 2017-05-01

**Authors:** Mary Clare Houlihan, Joseph D. Tariman

**Affiliations:** DePaul University School of Nursing and DePaul University, Chicago, Illinois

## Abstract

Despite widespread use of support groups in the breast cancer patient population, there are heterogeneous outcome measurements and inconsistencies in their perceived benefits. The purpose of this integrative literature review is to compare the efficacies of traditional and online support groups for breast cancer survivors through analysis of outcome measurements and determination of strengths and weaknesses. After examining the literature, it was found that online support groups are ideal for women who require additional support or who are unable to attend a traditional group. Alternatively, traditional support groups allow for discussion and support tailored to specific cultures and are especially beneficial when a breast cancer survivor is included in the process. These findings suggest that because both traditional and online support groups have unique roles in the psychosocial support of female breast cancer survivors, individual preferences and needs should be considered when determining which support groups will be beneficial.

Breast cancer is the most common cancer in women across all races and ethnicities in the United States (American Cancer Society [ACS], 2016; Centers for Disease Control and Prevention, 2015). In 2016, it is estimated that 246,660 new cases of invasive breast cancer were diagnosed and 40,890 deaths resulted from breast cancer (ACS, 2016). Despite its prevalence, breast cancer death rates have declined by 36% from 1989 to 2012, implying improved prognosis for those diagnosed (ACS, 2016).

Although breast cancer boasts a high survival rate due to the success of current treatments, there are still many challenges, side effects, and survivorship issues related to the disease processes and treatments, including body image concerns, psychological distress, psychosocial functioning, quality of life, sexuality, financial concerns, and fatigue (Miller, 2008; Sadler-Gerhardt, Reynolds, Britton, & Kruse, 2010; Shannonhouse et al., 2014). These side effects are severe and can have long-lasting consequences.

A 5-year study on breast cancer patients suggested that nearly half of the women demonstrated signs of depression, anxiety, or both 1 year after diagnosis, and 25% experienced depression, anxiety, or both up to 4 years after diagnosis (Burgess et al., 2005). Additional studies showed that 30% to 50% of breast cancer patients present with increased fatigue, which persists for up to 5 years after treatment (Björneklett et al., 2012a; Minton & Stone, 2008). These unwanted effects warrant a need to address the unique concerns of breast cancer survivors.

## SOCIAL SUPPORT GROUP

One way to address the needs of these survivors is through the use of a social support system. The concept of social support is defined as a collaborative exchange of information, emotions, and practical advice between donors and recipients (Bender, Katz, Ferris, & Jadad, 2013). A tangible application of this concept is face-to-face or in-person support groups, which will be defined as traditional support groups throughout this literature review. Traditional support groups emerged in the cancer patient population in the 1970s (Klemm et al., 2003) and have been continually used to improve the health and well-being of individuals with breast cancer over the past several decades (Bender et al., 2013).

In recent years, increased access to and popularity of the internet have led to the utilization of online social support for breast cancer survivors (Van Uden-Kraan et al., 2008). Like traditional support groups, online support groups are designed to provide an environment in which individuals can share experiences and exchange information, advice, and support (Griffiths, Calear, & Banfield, 2009). Online support groups have been praised because of their convenience, anonymity, and affordability (Klemm et al., 2003; Lepore et al., 2014). However, despite sufficient research on the role of online support groups in the breast cancer patient population, there are heterogeneous outcome measurements and mixed evidence of their efficacy (Lepore et al., 2014). In addition to the inconclusiveness of the efficacy of online support groups, there are few articles that compare the effectiveness of traditional and online support groups for this population.

The inconsistencies in the perceived benefits of the efficacy of online support groups as compared with traditional support groups warrant more research. This integrative literature review analyzes both traditional and online support groups for breast cancer patients. It also demonstrates a preferred avenue of support for breast cancer patients, which could promote improved quality of life and overall health in this population. Therefore, the purpose of this integrative literature review is to compare the efficacies of traditional and online support groups for breast cancer survivors through analysis of outcome measurements. From this comparison, strengths and weaknesses of both online and traditional groups are determined.

The following research questions were created to guide this study:

1. What are the overall outcomes of online support groups as compared with traditional support groups for breast cancer survivors?
2. Is one type of support group better suited for breast cancer survivors or a subset of this population than another?

## CONCEPTUAL FRAMEWORK

The Social Network Theory (SNT) serves as the conceptual framework for this article. The theory proposes that social interactions among individuals generate heterogeneous relationships with varying levels of supportiveness (Pierce, Sarason, & Sarason, 1991). The fundamental concept of the SNT is the network, which is defined as a set of individuals and a set of common ties that connect these individuals (Daly, 2010). Unlike a simple relational orientation, the SNT considers the incorporation of individuals in a web of relationships and the impact these relationships can have on a given individual’s opportunities (Daly, 2010). An important aspect of this theory is that it does not treat interactions between individuals in isolation; rather, it considers the pathways through which information flows and the indirect effects of interactions (Daly, 2010).

According to the SNT, both the information pathway and the effects of interactions form a structure, which is occupied by a particular individual. This structure determines the opportunities and obstacles an individual encounters, thus affecting the outcome of that individual’s experience in a certain network. Because the SNT accounts for both the structure and property of a given network, it will provide a framework for analyzing the outcomes of traditional and online support groups.

The SNT provides a framework that captures the multidimensional relationships among individuals in both traditional and online support group environments. The theory advocates for individuals to make appropriate and effective use of social support by engaging them in the identification of potential social supports groups (Kang’ethe, 2011). The SNT identifies potential support groups by contextualizing the structure, relationships, and outcomes of different support groups (Daly, 2010). By providing a context for how individuals interact within a given environment, the SNT offers better understanding of the outcomes of breast cancer survivors’ interactions in traditional and online support networks. Additionally, use of the SNT to understand the social interactions that connect individuals to others allows for evaluation and consideration of the social capital of traditional and online groups and the individual members who comprise them (Kang’ethe, 2011).

## METHODS

**Design**

An integrative literature review is a method that analyzes and synthesizes literature to provide a comprehensive understanding of a particular phenomenon or problem (Whittemore & Knafl, 2005). Therefore, this methodology was used to compare the outcomes of online and traditional support groups for breast cancer patients, because there are discrepancies in the perceived benefits of both groups due to heterogeneous outcome measures. Due to the varied outcome measures and the lack of an integrative review on this topic, the benefits and disadvantages of both online and traditional support groups for breast cancer patients are the focus of analysis and synthesis in this integrative literature review.

**Literature Search Strategies**

Three computerized databases were searched in this review: CINAHL, PsychInfo, and PubMed. The terms "breast neoplasm" and "support groups" were used to search both CINAHL and PsychInfo databases. The terms "breast neoplasm" and "self-help groups" were used to search the PubMed database. Additionally, "psychosocial support" was used as a search term in all three databases.

After initial searches using these terms, inclusion criteria to select only articles with the "support group" major headings were chosen in the CINAHL and PsychInfo databases. Inclusion criteria were used in PubMed to select articles with the MeSH terms "breast neoplasm" and "self-help groups." Among these articles, only peer-reviewed journals were chosen. Search criteria were then limited to articles that were published after 2005. Relevant abstracts were then chosen from each database, and duplicate articles were removed. Finally, articles were read and further examined to determine whether they address outcome measures for online breast cancer supports, traditional breast cancer support groups, or both. Articles that met these criteria were used for this integrative review. A review of the literature was performed, and articles were gathered based on selection criteria (Figure).

**Figure:**
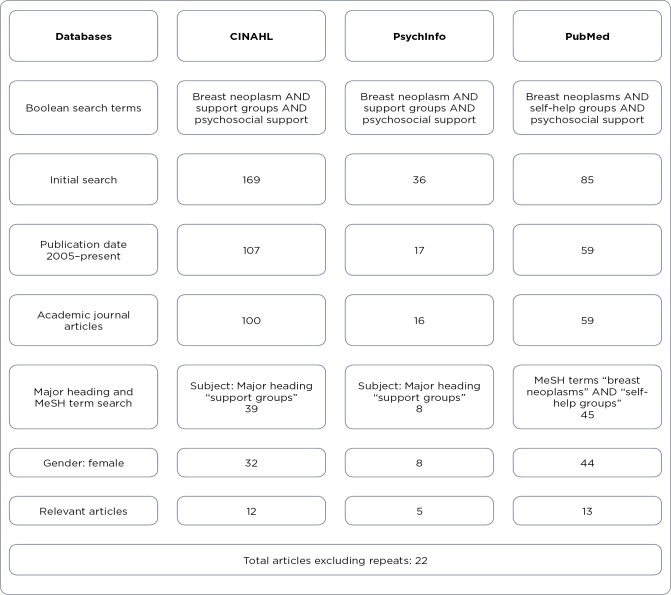
Process of inclusion and exclusion of studies used in this integrative review.

**Data Analysis**

The five-step integrative method by Whittemore and Knafl (2005) was used in this literature review. The steps for this method include: (1) problem identification; (2) literature search with inclusion and exclusion criteria; (3) data evaluation; (4) data analysis through extraction and reduction; and (5) presentation (Whittemore & Knafl, 2005). First, research questions were defined to guide this study and facilitate data extraction from primary sources. Next, appropriate primary sources were identified in the literature search step using three databases and several specific key terms. Data were then evaluated using methods and criteria to ensure they were authentic and appropriate. Following evaluation, data were analyzed to interpret the effectiveness of different cancer support groups and identify specific themes and patterns. Finally, conclusions were drawn from data analysis, and results were displayed in two tables (Tables 1 and 2). Additionally, the data integration process used to identify primary sources is displayed in the Figure.

**Table 1:**
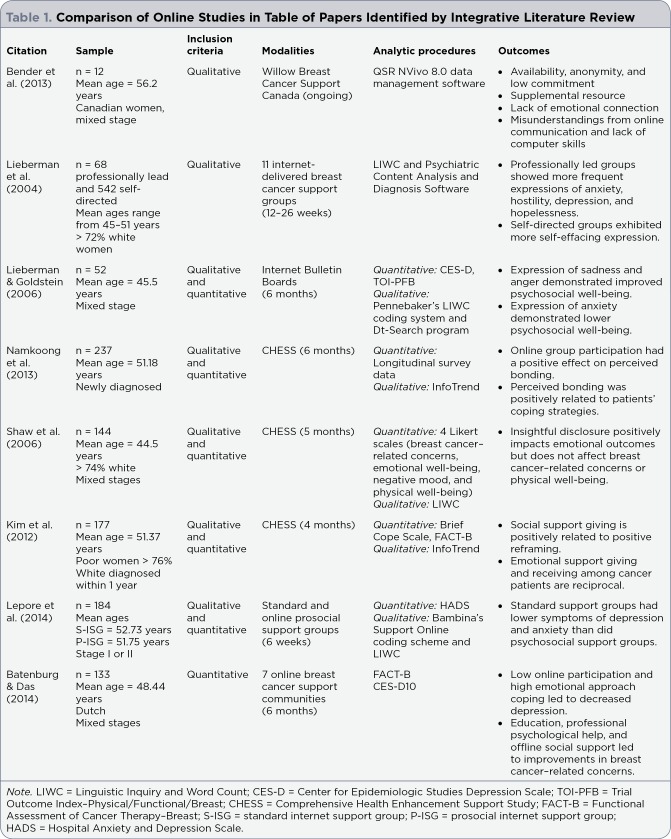
Comparison of Online Studies in Table of Papers Identified by Integrative Literature Review

**Table 2:**
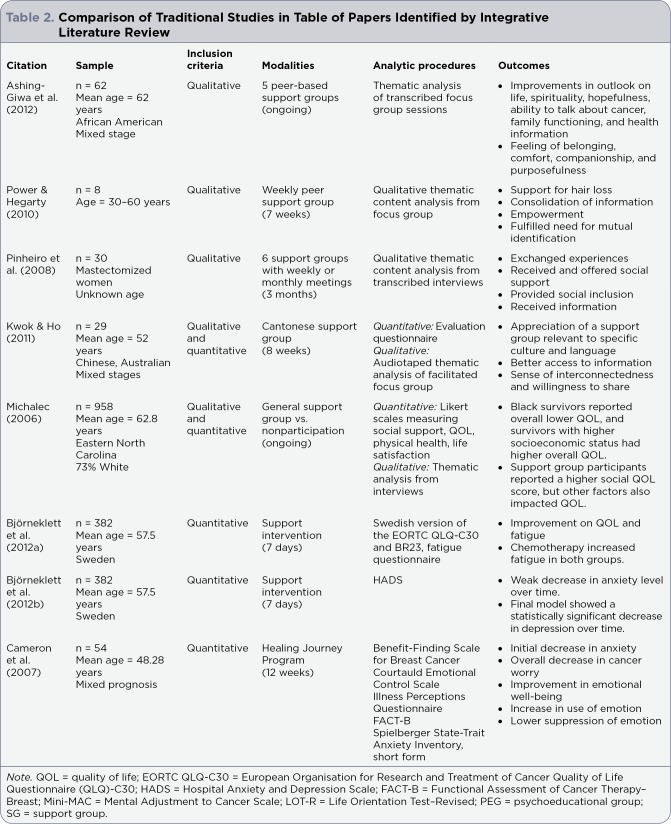
Comparison of Traditional Studies in Table of Papers Identified by Integrative Literature Review

**Table 2:**
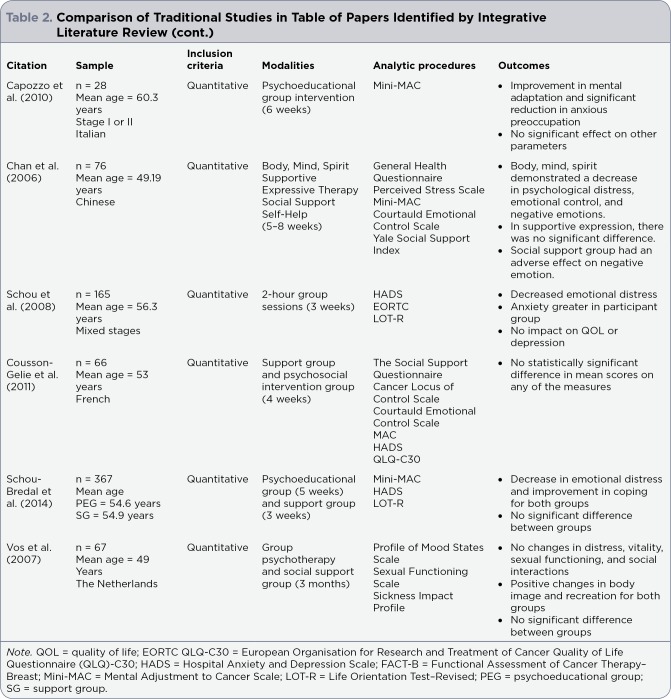
Comparison of Traditional Studies in Table of Papers Identified by Integrative Literature Review (cont.)

## RESULTS

Articles used in this study are grouped according to program design (online or traditional) and inclusion criteria (qualitative or quantitative). Qualitative analyses were performed in two of the online support group articles; quantitative analysis, on one; and mixed analyses, on the remaining five articles. For traditional support group articles, three articles used qualitative analyses, nine used quantitative analysis, and two used mixed analysis.

Research questions that guided this review were answered to compare overall outcomes and determine unique group features that might benefit a specific population of breast cancer survivors. It was found that online groups allow for user anonymity, flexibility, and low commitment, making them beneficial for women who require additional support or are unable to attend a traditional support group due to geographic or time constraints. Traditional groups have proven effective because they can provide culturally competent and linguistically appropriate support tailored to specific communities of breast cancer survivors. Summary findings of the articles analyzed in this review are found in Tables 1 and 2.

## DISCUSSION

Due to their unique features, online and traditional support groups can offer important resources for women with breast cancer. It has been demonstrated that both types of support groups can positively impact well-being and decrease anxiety levels (Cameron, Booth, Schlatter, Ziginskas, & Harman, 2007; Lieberman & Goldstein, 2006). However, each group has inherent strengths and weaknesses that impact its effectiveness in different populations of breast cancer survivors. Online support groups might be useful for women who require supplemental support, but these groups do not necessarily compensate for the lack of support from relatives and deteriorated health status. Traditional groups can be used to provide culturally competent and linguistically appropriate support for women but might not be helpful for women who are physically unable to attend a group (Ashing-Giwa et al., 2012; Kwok & Ho, 2011).

As predicted by the SNT, because both online and traditional groups have inherently different structures and information pathways, there are distinct social interactions and unique relationships formed between individuals in each group. This results in different outcomes due to an individual’s experience in a certain social environment and provides perspective on member interactions and expression in these separate domains. This framework provides an understanding of how social environment and interactions impact the outcomes of both types of support groups throughout this literature review.

## OUTCOMES OF ONLINE AND TRADITIONAL SUPPORT GROUPS

The first objective of this review was to compare the overall outcomes of online and traditional support groups. As mentioned previously, it is necessary to consider the information pathway and social interactions to understand and compare group outcomes.

Studies on both online and traditional groups focused on emotional exchanges and individual interactions to measure their effectiveness. Five studies focused specifically on expression of emotions in online groups and the impact a professional leader has on outcomes (Lepore et al., 2014; Lieberman, Golant, Winzelberg, McTavish, & Gustafson, 2005; Lieberman & Goldstein, 2006; Namkoong et al., 2013; Shaw, Hawkins, McTavish, Pingree, & Gustafson, 2006).

As demonstrated by Namkoong et al. (2013), participating in supportive exchanges with others in online support groups leads to a positive effect on perceived bonding between individuals, which can improve coping strategies. In contrast, additional findings propose that lack of both supportive exchange and communication about an individual’s cancer experience in online groups may serve as a negative stressor on the body adversely affecting physical well-being (Shaw et al., 2006).

In addition to the negative impact lack of communication has on an individual, it has been demonstrated that professionally led online groups show decreased psychological well-being when there are frequent expressions of anxiety, depression, and hostility as well as fewer positive emotions than self-led online groups (Lieberman et al., 2004). According to a similar study, self-led groups demonstrated more self-effacing emotions of sadness and anger, which resulted in improved psychosocial well-being for individuals (Lieberman & Goldstein, 2006).

Professionally led online groups that have an involved group leader may increase anxiety and unintentionally lead participants to be acutely self-aware in their responses and hold back feelings in fear of upsetting others (Lepore et al., 2014). In contrast, self-directed groups demonstrate more emotional expression than groups with a leader, suggesting that participants had fewer concerns about burdening others with their cancer-related concerns due to the unique chance to talk freely with empathic others in an online self-help group (Lepore et al., 2014). Based on these findings, self-led online support groups might prove beneficial to women with breast cancer because they encourage free exchange of feelings and emotional disclosure among participants. Professionally led online groups, however, may provide a more constructive forum that could allow for individuals to engage in more directed, therapeutic conversations with a trained leader.

Similar to with online groups, emotional exchange of experiences between participants is important to the success of traditional groups (Pinheiro, da Silva, Mamede, & Fernandes, 2008). As mentioned previously, studies of online support groups with an involved facilitator lead to more frequent negative outcomes for individuals (Lepore et al., 2014; Lieberman et al, 2004; Lieberman & Goldstein, 2006). Unlike online groups, however, an active facilitator who is also a cancer survivor in traditional groups has been shown to empower participants because of the mutual identification this individual provides (Power & Hegarty, 2010). Having a breast cancer survivor as a facilitator in traditional groups has been shown to be instrumental in providing participants with the necessary skills needed to cope with the daily problems associated with a breast cancer diagnosis (Power & Hegarty, 2010).

In addition to considering information pathways and social interactions among group participants, it is necessary to consider the type of data analysis performed to determine outcomes. Literature on traditional groups throughout this review includes a variety of qualitative, quantitative, and mixed analyses. Most studies of online support groups, though, use mixed qualitative and quantitative analysis, which provides multiple avenues through which the success of a group can be measured. A majority of these studies use diagnostic software to perform a thematic analysis on group discussions in conjunction with quantitative questionnaires. Software analysis often allows for extrapolation of common themes in online discussions and can provide an integral understanding of the type of support provided.

Although most online studies used a mixed approach, one study utilized solely a quantitative approach to measure emotional coping, emotional well-being, and depression. Like many studies that utilize only a quantitative approach, this study did not find any correlation between improvement in depression or emotional well-being after use of an online support group (Batenburg & Das, 2014). This finding suggests that other factors outside of the online environment may affect outcome measures and therefore may confound some of the data. For example, upon further analysis of data, Batenburg and Das (2014) found that patients who received support from family and friends reported a higher well-being than those without support.

Additionally, a study on traditional support groups did not produce any statistically significant data; however, after the intervention was complete, women were interviewed, and those who participated in the group psychotherapy stated they had learned more to express themselves and exhibited increases in the use of emotion-regulation strategies and perceived control after the intervention (Cameron et al., 2007). Future studies should consider utilizing a mixture of qualitative and quantitative measures for data analysis. Although quantitative data can provide measurable data and include covariates that affect measurement outcomes, they can also fail to delve into more informative data, which could be obtained using a combination of both qualitative and quantitative studies.

As mentioned previously, several studies on traditional support groups used strictly quantitative measures to analyze data. These measures included the European Organisation for Research and Treatment of Cancer (EORTC) Quality of Life Questionnaire (QLQ)-C30, QLQ-BR23, Hospital Anxiety and Depression Scale (HADS), Functional Assessment of Cancer Therapy–Breast (FACT-B) scale, Life Orientation Test–Revised (LOT-R), Courtauld Emotional Control Scale, and Mini-MAC (Mental Adjustment to Cancer) scale; they were used to determine the effectiveness of a group after it was implemented for only a short period (Björneklett et al., 2012a, 2012b; Capozzo, Martinis, Pellis, & Giraldi, 2010; Schou, Ekeberg, Karesen, & Sorensen, 2008; Bredal et al., 2014; Vos, Visser, Garssen, Duivenvoorden, & Haes, 2007).

Most studies resulted in weak trends or nonsignificant data. However, two studies demonstrated significant, positive change in participants after the use of a traditional support group. Capozzo and colleagues (2010) demonstrated an improvement in mental adaptation and significant reduction in anxious preoccupation in participants, and Vos and colleagues (2007) measured a positive change for body image and recreation. These significant data may be due to a longer time frame of intervention (6 weeks and 3 months) and the smaller sample sizes of these groups (n = 28 and 67) compared with the other studies. A longer time frame for these two groups would allow participants to acclimate to the group, and a smaller sample size would result in less variation between different support groups within the study. The other four studies that did not produce any significant data included shorter time frames (7 days to 5 weeks) and larger sample sizes (165–382 participants), allowing for more variation between individual groups within a study. Despite the many obstacles produced from analysis of short-term traditional support group interventions, further study of these groups may illustrate how a short-term intervention might benefit individuals during a difficult period, such as when patients are newly diagnosed or receive adjuvant chemotherapy or radiotherapy.

Methodologic flaws used in studies can confound the actual benefits of support group participation on quality of life as it relates to different types of breast cancer survivors (Michalec, 2006). Therefore, it is important to consider covariates and demographics when analyzing or comparing data. For example, one study found that being African American was found to have a significantly negative effect on social quality of life (Michalec, 2006). Additionally, considering the mean age of women who participate in online support groups is necessary, because lack of computer skills in middle-aged and older adult populations could account for discontent with online support groups. Finally, it is important to consider different factors and social determinants when evaluating data and suggesting support groups for specific populations, because they affect how an individual perceives support.

## UNIQUE GROUP FEATURES TO DETERMINE USER PREFERENCE

The second objective of this review was to determine whether one type of support group is better suited for breast cancer survivors or a subset of this population than another. As previously mentioned, support groups have unique features that allow them to provide specialized care and resources for breast cancer survivors. Because online groups are accessible anywhere there is an internet connection, they can work as a supplement to traditional face-to-face groups and can provide support for those who wish to remain anonymous or are unable to attend an in-person meeting (Bender et al., 2013). Due to the lack of physical and time constraints in online groups, exchanges between individuals can lead to more fluid, less restrictive conversations than in traditional support groups, which can lead to higher levels of emotional support from others and fewer breast cancer–related concerns (Kim et al., 2012). However, despite their flexibility and usefulness, users of online support groups have also been shown to lack emotional connections and to have increased misunderstandings due to lack of face-to-face contact or poor computer skills (Bender et al., 2013).

Since traditional support groups require face-to-face communication and are often community-based, they allow for discussion and support tailored to specific cultures, which is not always feasible with an online group (Ashing-Giwa et al., 2012; Kwok & Ho, 2011). Ashing-Giwa et al. (2012) found that African American breast cancer survivors preferred a culturally sensitive forum that responds to their unique psychosocial, spiritual, physical, and informational needs. Peer-based groups facilitated by an African American breast cancer survivor allow members to be more comfortable by relating to other members with similar cancers and cultural experiences (Ashing-Giwa et al., 2012). Two other studies found that providing culturally competent support and resources for Chinese breast cancer survivors resulted in improved access to information and a sense of interconnectedness among individuals (Kwok & Ho, 2011; Chan et al., 2006).

In contrast, Cousson-Gélie, Bruchon-Schweitzer, Atzeni, and Houede (2011) found that few breast cancer survivors in France wished to attend a psychological support group. The few who did benefit were the most vulnerable, with worse initial quality of emotional life and smaller social network, suggesting that group therapy may not be well accepted by a majority of patients in this setting (Cousson-Gélie et al., 2011). In addition to considering structure, information pathway, and interactions in a group, it is necessary to consider culture, beliefs, and support systems to provide supportive resources for breast cancer survivors.

## STUDY LIMITATIONS

Although this review provides insight into outcomes of breast cancer support groups, there are several limitations to consider. To begin, since only psychosocial studies were chosen for this review, a selection bias may exist. Despite exclusively including studies on breast cancer in women, this review did not consider the stage or progression of different breast cancers during analysis. This is important to consider because disease progression could impact outcome measurements and create inconsistencies when comparing studies.

Another limitation to this study includes the heterogeneity among support group types. Although the 22 different studies focused on psychosocial support, each support group is unique and therefore inherently difficult to compare with other support groups.

A final limitation of this review is the lack of studies that consider both online and traditional support groups. Although studies exist that compare online and traditional groups, the search criteria used in this review did not include any of them.

## IMPLICATIONS FOR NURSING PRACTICE

These findings underscore the importance of considering individual differences in dealing with illness when examining health outcomes of support communities. No support community provides a blanket solution for individuals, because not all support groups compensate for the potential needs of all breast cancer survivors (Bender et al., 2013). Implications for nursing practice include considering the individual’s wants and needs when recommending a breast cancer support group. An individual’s schedule, literacy, and access to the Internet and transportation should be assessed when recommending a group. An individual’s psychosocial needs should also be considered to determine which environment would best support the individual. In addition to accounting for individualized needs while recommending support groups, providers should consider the timing for breast cancer support group recommendations. Introducing individuals to a support group at the time of diagnosis could allow them to obtain support almost immediately or allow them to access a group at their own pace. Reinforcing their options throughout their treatment and survivorship phases could also optimize an individual’s support throughout their diagnosis.

These recommendations can be applied more broadly to nursing practice when counseling patients on other treatment options or health care decisions. Additionally, the recommendations from this study stress that advanced practice nurses and other advanced practice providers must be active listeners and culturally competent care providers to address the needs of their patient population.

## CONCLUSION

This review compared the outcomes of both online and traditional breast cancer support groups through an integrative analysis of articles. Because both traditional and online support groups have unique roles in the psychosocial support of female breast cancer survivors, individual preferences and needs should be considered when determining which support groups will be beneficial. Advanced practitioners should invest in future studies focusing on online support due to improvements in online access and internet knowledge in the past several years. Additionally, studies should focus on how online support can best be used to help individuals from different cultures and globally underserved communities.
